# Individual modelling of haematotoxicity with NARX neural networks: A knowledge transfer approach^☆^^☆☆^^☆☆☆^^☆☆☆☆^

**DOI:** 10.1016/j.heliyon.2023.e17890

**Published:** 2023-07-05

**Authors:** Marie Steinacker, Yuri Kheifetz, Markus Scholz

**Affiliations:** a Center for Scalable Data Analytics and Artificial Intelligence (ScaDS.AI) Dresden/Leipzig, Leipzig University, Germany; b Leipzig University, Medical Faculty, Institute for Medical Informatics, Statistics and Epidemiology (IMISE), Germany; c Leipzig University, Faculty of Mathematics and Computer Science, Germany

**Keywords:** Recurrent neural networks, System identification, Haematopoiesis, Precision medicine, Transfer learning

## Abstract

Cytotoxic cancer therapy often results in dose-limiting haematotoxic side effects. Predicting an individual's risk is a major objective in precision medicine of cancer treatment. In this regard, patient heterogeneity presents a significant challenge. In this paper, we explore the use of hypothesis-free machine learning models based on recurrent nonlinear auto-regressive networks with exogenous inputs (NARX) as an approach to achieve this goal. Also, we propose a knowledge transfer approach to ameliorate the issue of sparse individual data, which typically hampers learning of individual networks. We demonstrate the feasibility of our approach based on a virtual patient population generated using a semi-mechanistic model of haematopoiesis and imposing different cytotoxic therapy scenarios on it. Employing different techniques of model optimisation, we derive robust and parsimonious individual networks with good generalisation performances. Moreover, we analyse in detail possible factors influencing the generalisation performance. Results suggest that our transfer learning approach using NARX networks can provide robust predictions of individual patient's response to treatment. As a practical perspective, we apply our approach to individual time series data of two patients with non-Hodgkin's lymphoma.

## 1 Introduction

Cytotoxic cancer therapies frequently result in severe, often dose-limiting haematotoxic side effects [1]. There is a high heterogeneity between patients [2] so that possibly a small group of patients with higher sensitivity to cytotoxic drugs imposes treatment constraints on a larger patient population for safety reasons. Predicting a patient's haematologic response to treatment and adapting the therapy accordingly is therefore a major task in precision-medicine concepts of cancer treatment. Since pre-therapeutic risk models are not reliable, the idea is to improve individual predictions based on the observed data during the first therapy cycles. To solve this task, statistical risk models [1], semi-mechanistic models of bone marrow haematopoiesis [3], [4], [5], [6] and comprehensive mechanistic models [7], [8], [9] were proposed. The latter showed decent success in supporting individualised treatment decisions [9]. However, there are subsets of patients exhibiting irregular dynamics, which could not be predicted by mechanistic models [9].

In this paper, we examine whether hypothesis-free machine learning models could be an alternative to semi-mechanistic modelling. We explore a knowledge transfer approach to apply these machine learning models to individual patient time series data and investigate the embedding of a semi-mechanistic model in this framework. In detail, we apply recurrent neural networks (RNNs) based on nonlinear auto-regressive exogenous (NARX) models to describe the highly nonlinear dynamics of haematopoiesis under chemotherapy. This is motivated by the universal mapping property of neural networks showing that arbitrary complex functions can be approximated [10], [11].

RNNs already have been applied to various bio-medical tasks, for example in predicting clinical events [12] or modelling the progression of Alzheimer's disease [13]. NARX networks differ from standard RNNs, as they use an additional exogenous control input to predict the systems evolution with time. The general performance of NARX networks on time series data has been tested extensively in the past [14], [15], [16], showing that NARX networks outperform standard neural network based predictors.

To cope with the relative sparsity of individual patient data and to improve training speed, we implement a transfer learning process. The concept of transfer learning is commonly used in machine learning problems [17], where only sparse data or sparse training resources are available, but a large general model or data pool for a similar application already exists. We test this framework based on a virtual patient population under different treatment scenarios. We generate the virtual patient data with the help of a commonly used semi-mechanistic model of Friberg et al. [3], where inter-individual variability is imposed by different settings of biological parameters. For each virtual patient, we train a personalised prediction model using NARX neural networks and analyse prediction performance based on therapy scenarios not used for model training. We employ a combination of several model optimisation methods to avoid over-fitting and to derive robust parsimonious individual networks. We demonstrate that our approach is feasible to learn individual models with good prediction performances on a limited set of data allowing translation into practical applications. Finally, we show an envisaged perspective for real world applications of our approach by predicting leukocyte dynamics of two selected patients with non-Hodgkin's Lymphoma. Prediction performances were compared between our approach and the semi-mechanistic model.

## 2 Methods

To develop our approach, we first establish a virtual data set of patients under cytotoxic therapies by simulating a semi-mechanistic model of haematopoiesis. Then, we implement our Neural Network (NN) approach to learn individual patient time courses with a knowledge transfer process.

### 2.1 Virtual patient population

Here, we briefly describe the semi-mechanistic model of haematopoiesis we use to simulate our population of virtual patients.

#### 2.1.1 The semi-mechanistic model

A simple semi-mechanistic ordinary differential equations model of haematopoiesis also considering cytotoxic drug applications was proposed by Friberg et al. [3], [4]. It is based on the simple assumption of splitting bone marrow cell lines into a proliferating and a maturing compartment. The proliferating compartment is susceptible to chemotherapy. The maturing compartments essentially impose a time delay between the proliferating compartment and the circulating cell compartment which can be measured by blood samples. Also, there is a feedback loop signalling the need of circulating cells to the proliferating compartment. If the circulating compartment is below normal, proliferation is increased above normal and vice versa.

To describe time courses of different patients, inter-individual heterogeneity of model parameters is assumed. Three of these parameters are unrelated to therapy: The baseline value of circulating white blood cells $Circ_{0}$, the transit time of bone marrow cell stages *MTT* representing the maturation process from early stem cells to mature circulating cells and a parameter *γ* controlling the strength of the feedback mechanism of the number of circulating cells on the stem cell proliferation compartment of the model.

Inter-individual heterogeneity is also assumed for up to two therapy-related parameters. During chemotherapy, the model assumes a reduction of the proliferating activity by a factor of $1-E_{drug}$. At this, $E_{drug}$ is a Michaelis-Menten function depending on the chemotherapy concentration Conc, Equation (1):

(1) $$E_{drug}=E_{max}\frac{Conc}{EC_{50}+Conc}.$$

The two therapy related parameters in Equation (1) are the maximum therapy effect $E_{max}$ and the chemotherapy concentration corresponding to the half-maximum effect $EC_{50}$. Here, we assume inter-individual variability (iiv) only for the first parameter. The parameter $EC_{50}$ is fixed to a value of 5.2 [3], as it is difficult to distinguish its impact from that of *γ* and $E_{max}$ according to our analyses.

The model is described by the following differential equations, Equations (2) to (6), [3]:

(2) $$\frac{d}{dt}Prol=k_{tr}\cdot Prol\cdot (1-E_{drug}){\left(\frac{Circ_{0}}{Circ}\right)}^{\gamma }-k_{tr}\cdot Prol$$

(3) $$\frac{d}{dt}T_{1}=k_{tr}\cdot Prol-k_{tr}\cdot T_{1}$$

(4) $$\frac{d}{dt}T_{2}=k_{tr}\cdot T_{1}-k_{tr}\cdot T_{2}$$

(5) $$\frac{d}{dt}T_{3}=k_{tr}\cdot T_{2}-k_{tr}\cdot T_{3}$$

(6) $$\frac{d}{dt}Circ=k_{tr}\cdot T_{3}-k_{tr}\cdot Circ.$$

**Figure 1:**
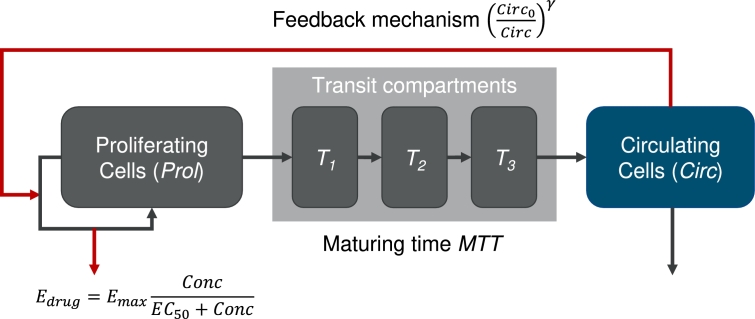
**Model diagram of the semi-mechanistic model of haematopoiesis** according to Friberg et al. [3], including an inhibitory effect of chemotherapy via the function *E*_*drug*_ depending on the drug concentration *Conc*. While black arrows represent first order transitions, red arrows represent feedbacks and actions.

A diagram depicting the semi-mechanistic model and its regulations is shown in Fig. 1.

#### 2.1.2 Virtual patient data

We use this model to simulate data for a population of patients. We only consider neutrophil dynamics and therapies with the cytotoxic drug etoposide with varying dosing and timing schedules. Etoposide is considered since a pharmacokinetic and -dynamic model was readily provided in [3] and was attached to the semi-mechanistic haematopoiesis model. The elimination half-life of etoposide is given as  [18]. We simulate etoposide applications as a three-day continuous infusion. For all virtual patients, we assume the same volume of distribution of the drug, namely  [19].

Heterogeneity of our virtual patient population is generated by choosing different values for the iiv parameters mean transit time *MTT* and feedback strength *γ*. The steady state parameter $Circ_{0}$ is a scaling factor, which does not affect the dynamics. Therefore, we set it constant  according to the population average [3]. Heterogeneity of the therapy effect is generated by assuming different values for parameter $E_{max}$.

We choose values of the iiv parameters in physiological ranges as identified in [3]. These ranges are provided in Table 1. The chosen parameter ranges span a hypercube in the parameter space, and a single virtual patient is represented by a point in this hypercube. We sample from the hypercube in the following way: We consider eight equidistant values in the directions of *MTT* and *γ*. Regarding $E_{max}$, we consider eight points equidistant at the log-scale. Thus, we consider a total of 512 parameter combinations.

**Table 1:** **Ranges of parameters assumed to be heterogeneous in our virtual patient population.** We consider eight equidistant values in the provided ranges of *MTT* and *γ*. Regarding *E*_*max*_, we consider eight points equidistant at the log-scale of the provided range, i.e. a total of 512 parameter combinations were analysed.

Parameter	Meaning	Min.	Max.
*γ*	Feedback strength	0.1	0.25
*MTT*	Maturation time		
*E*_*max*_	Maximum therapy effect	0.785	3.14

**Figure 2:**
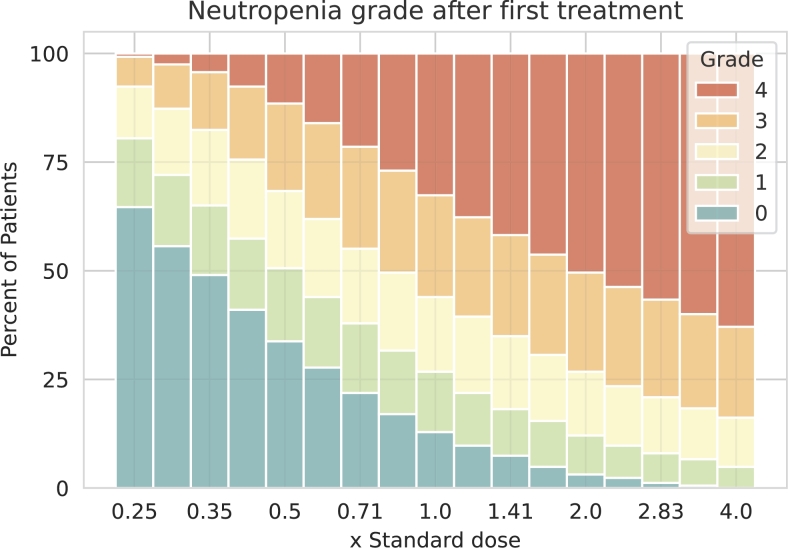
**Distribution of neutropenia grades in our virtual patient population after a single chemotherapy application.** For each dose level given as fractions of the standard dose, we simulated the distribution of resulting neutropenia grades in our virtual patient population. Neutropenia grades are distinguished by colour, from blue for neutropenia grade 0, to red for neutropenia grade 4. We observed a realistic spectrum of neutropenia grades in dependence on chemotherapy dosage.

To explore our virtual patient population, we determine the severity of neutropenia after a single administered dose. We classify severity of neutropenia in grades as in [20]: grade 0: ; grade 1: ; grade 2: ; grade 3: ; grade 4: . In our treatment simulation, drug dosage is varied in between one fourth and four times the standard total dose of 375 mg/m^2^. Resulting distributions of neutropenia grades is shown in Fig. 2. We conclude that our virtual patient population reflects a realistic spectrum of neutropenia grades in dependence on chemotherapy dosage (compare to [1]).

We define a total of 18 different therapy scenarios. These therapy scenarios are later considered for training (five therapy scenarios), validating (two) and testing (11) our neural network approach. To define a scenario, we randomly choose six starting days of therapy with a minimum distance of one week. For each administration, the drug dosage is also chosen randomly between one fourth and four times the standard total dose of 375 mg/m^2^. Resulting scenarios are provided in supplemental Table S1, where we also provide the software code to generate the therapy dates and dosages.

**Figure 3:**
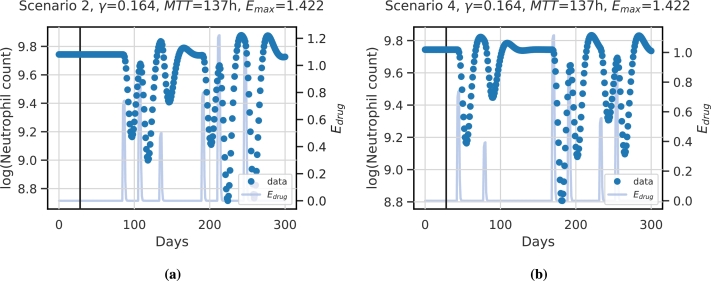
**Example dynamics of virtual patient data:** We present the dynamics of neutrophils (blue dots, left y-axis) of a specific patient with iiv parameters *γ* = 0.164,  and *E*_*max*_ = 1.422 under **(a)** therapy scenario 2 and **(b)** therapy scenario 4. The horizontal line corresponds to 28d according to the assumed memory of the model. Dynamics of scenario-specific cytotoxic drug action *E*_*drug*_ (light blue curves, right y-axis) is also displayed.

Then, we apply these therapy scenarios to each of the 512 virtual patients, i.e. we simulate the dynamics of the given 18 therapy scenarios for every virtual patient parameter setting. The individual time series data of neutrophils we simulate consist of 300 consecutive days. Example simulations of model scenarios for a specific patient are depicted in Fig. 3. In Fig. 3a the simulation for scenario 2 is shown, and in Fig. 3b the simulation for scenario 4.

We implement the differential equations of the semi-mechanistic model in Python and solve the differential equation system with the solve_ivp function using the LSODA solver provided by SciPy [21].

### 2.2 Neural network approach to learn individual patient dynamics

**Figure 4:**
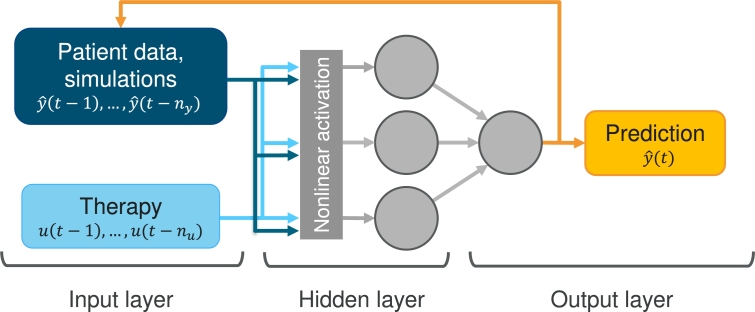
**Example scheme of a NARX network architecture with application to patient data:** An example network with one hidden layer with three neurons is shown. The data *u*, in our case the administered therapy, serve as input to the system. Different therapy scenarios can be considered with the same model by adapting the therapy input.

We aim at learning our virtual patient data using a NARX neural network. NARX neural networks are an adaption of so-called nonlinear auto-regressive models with exogenous inputs, with the auto-regressive model being a neural network. In contrast to most recurrent network models, the recurrent connection in NARX networks is formed by a so-called tapped delay line [22] from the output neuron of the network to the input neurons, and not from the hidden states. The nonlinearity in NARX networks stems from a nonlinear neuron activation function. The general formula describing NARX models is as follows, Equation (7),

(7) $${\overset{ˆ}{y}}_{t}=F\left({\overset{ˆ}{y}}_{t-1},\ldots ,{\overset{ˆ}{y}}_{t-n_{y}},u_{t-1},\ldots ,u_{t-n_{u}}\right),$$

where $n_{y}\in N$ and $n_{u}\in N$ are the memory sizes for the system output and exogenous input respectively; $u_{t}\in R^{x}$ is the exogenous input to the system, ${\overset{ˆ}{y}}_{t}\in R^{z}$ is the system output at discrete time *t*, and F is a nonlinear function of the input (*u*) and the output (*y*) variables. In our case, this function is represented by a neural network. For the first $n_{y}$ time points, no network outputs are available. Thus, respective data points $y(1),\ldots ,y(n_{y})$ are used as input. The NARX neural network architecture is illustrated in Fig. 4, including our application to the neutrophil time series data of our virtual patients. The task is to describe these complex dynamics with a sparse individual network. The therapy function $E_{drug}$ serves as external input *u* to the network. The output of the network corresponds to the neutrophil counts of our virtual patients.

We base our approach on a NARX with a single hidden layer consisting of multiple neurons with nonlinear activation functions. Hyperparameters determining our network architecture are the number of neurons of the hidden layer, the size of input memory of the endogenous feedback $n_{y}$ and the therapy $n_{u}$. Further regularisation parameters are discussed in the next section. We tested commonly used activation functions (sigmoid, tanh, linear, ReLu) via hyperparameter optimisation on the virtual patient data. The standard sigmoid activation performed best. From the hidden layer, the processed information is passed to a single output neuron with linear activation. Optimisation is pursued by minimising the Bayesian Information Criterion (BIC) [23], described in detail in the next section. We chose the number of neurons in the hidden layer to lie between two to twelve, and the number of time points memorised between fourteen and 42 days. We utilise the tool Tune [24] with a random search algorithm for optimisation.

### 2.3 Learning approach

In this section, we provide details of our learning approach, which is based on transfer learning. More specifically, we learn the NARX framework based on the therapy scenarios of an index patient and update the weights based on the therapy data of a new patient represented by a new setting of our iiv parameters. In our hands, this type of transfer learning was much less compute intensive than the alternative of training a new network for each patient from scratch. Moreover, it better resembles the situation in practice where large patient data sets are available while the data situation for a single patient is sparse.

The parameter configuration of the index patient is γ=0.33, , $E_{max}=1.57$. While *MTT* and $E_{max}$ are within the parameter ranges described in Table 1, the parameter *γ* is chosen above the maximum value of the virtual patient population. The reason is that a high feedback value *γ* results in more extreme dynamics from which we expect that it is easier to infer less extreme patients.

#### 2.3.1 Measures to ensure model parsimony

Overfitting is a well-known problem of training neural network based models resulting in inferior prediction performance for new input scenarios. We apply the BIC to ensure model parsimony. We also apply magnitude pruning of connections between neurons, i.e. all weights lower than a certain threshold are set to zero [25]. During model training, pruning is controlled by the BIC of the model on training and validation data sets. We prune iteratively as long as the BIC improves. The pruning schedule is explained in detail in the next section. In our situation, the BIC formula is as follows, Equations (8) to (9),

(8) $$\text{BIC}(\{w\})=2nLL(\{w\})+k\ln(N_{tr}+N_{val}),$$

(9) $$nLL(\{w\})=\frac{1}{2}\sum_{t\in tr}{\left(\frac{\overset{ˆ}{y}(t)-y(t)}{\sigma }\right)}^{2}+\frac{1}{2}\sum_{t\in val}{\left(\frac{\overset{ˆ}{y}(t)-y(t)}{\sigma }\right)}^{2}+(N_{tr}+N_{val})\ln(\sigma ).$$

Here, *w* corresponds to the free parameters of the network, *nLL* is the negative log-likelihood, *tr* and *val* refer to time points in the training and validation set respectively, $N_{tr},N_{val}$ to the number of time points and *σ* is the residual error. The target data value at time *t* is denoted by y(t) and the predicted value is denoted by $\overset{ˆ}{y}(t)$. The value *k* refers to the number of free parameters that should be as small as possible. In our case, it is the number of non-zero weights in the model, as the number of hidden nodes was kept fixed for all individual models for comparability reasons. We assume that the errors are normally distributed. Then, the residual error can be estimated as, Equation (10),

(10) $$\sigma =\sqrt{\frac{\sum_{t\in tr}{\left(\overset{ˆ}{y}(t)-y(t)\right)}^{2}}{N_{tr}}},$$

and the negative log-likelihood can be estimated as, Equation (11),

(11) $$nLL(\{w\})=\frac{1}{2}N_{tr}+\frac{1}{2}\sum_{t\in val}{\left(\frac{\overset{ˆ}{y}(t)-y(t)}{\sigma }\right)}^{2}+(N_{tr}+N_{val})\ln(\sigma ).$$

To combat over-fitting, we use weight regularisation, sometimes also called weight decay [26]. Here, an additional weight dependent term is added to the data-dependent objective function during training. Traditionally, L2-regularisation is used, as it constraints the growth of weights. As we want to minimise the number of weights to obtain a sparse network in combination with pruning, we opt for a regularisation with the root of the weights, Equation (12),

(12) $$L=L_{data}+\alpha ⁎\sum_{i}\sqrt{\left|w_{i}\right|},$$

where L is the objective function of the network minimised in training, $w_{i}$ are the weights of the network and *α* is a tuning constant controlling regularisation strength. With this weight regularisation, small non-influential weights will be driven to zero and pruned faster. In hyperparameter optimisation, we optimise *α* between $10^{-1}$ and $10^{-7}$.

#### 2.3.2 Model training

In this section, we explain the model training for a single patient represented by a point in our parameter hypercube. Then, we introduce our approach of transferring the network architecture between hypercube points to obtain a class of models for the virtual patient population.

The network weights for the index patient are initialised randomly with zero mean and a standard deviation of 0.5. The network is then trained on the training data sets of the index patient with the “Adam” algorithm [27] as long as the objective function of the validation data sets improves. Then, we train again with iteratively increasing the threshold for magnitude pruning. The pruning threshold $\delta_{k}$ of the pruning step *k* is calculated as follows, Equation (13),

(13) $$\delta_{k+1}=1.1⁎\delta_{k}$$

All weights with absolute values smaller than $\delta_{k}$ are set to zero. The initial pruning threshold $\delta_{0}$ is set to 0.001. Pruning stops when the BIC does not improve anymore. The model of the second to last pruning step has the best BIC and is chosen as the training outcome.

Transfer learning is based on the successfully trained model of the index patient. Using the data of the five training scenarios, we perform the training of another patient represented by another point in the hypercube using the model weights obtained for the index patient to start with. Again, we use the BIC to control the progress of the training. Finally, we use the data of the eighth to eighteenth therapy scenarios to explore the prediction performances of the learned individual models, by comparing simulated results with the data.

### 2.4 Implementation

We logarithmize the time series data of neutrophils and standardise training data of each patient to zero mean and unit variance using the StandardScaler implementation by scikit-learn [28]. Then, we apply the obtained scaler function to the validation and testing scenarios. The function $E_{drug}$ is scaled to lie in between zero and one. Input scaling is necessary for the input of networks with a sigmoid activation function.

We implement the neural network models with Python. Existing implementations of NARX networks [29], [30] lack some fine-tuning capabilities required to apply our approach to sparse individual data. Therefore we decided to implement a class of NARX networks with TensorFlow [31] and Keras [32].

NARX networks can be trained in two different configurations: parallel, using regressed output as input, or series-parallel, using the true data as input. To avoid unstable network predictions for long simulations, we choose to train our networks in parallel configuration, as this results in more robust networks.

## 3 Results

We first present the learned architecture of our networks. Afterwards, we explore the learned virtual patients dynamics for the different therapy scenarios and analyse the network performance and size. Finally, we examine possible correlations between the trained networks properties and the virtual patient configuration.

### 3.1 Structure of the trained network

For comparability of scenarios, we keep the general network structure constant. Hyperparameter optimisation results in sigmoid activation of the hidden layer, four hidden neurons ($N_{n}=4$), a memory of $n_{y}=21$ days of previous network outputs and $n_{u}=28$ days of previous therapy inputs. Without pruning, this configuration results in networks with 200 trainable weights. We find α=0.0003 to be the optimal regularisation strength.

### 3.2 Learning of individual time series

First, we learn our index patient characterised by a parameter configuration resulting in particularly strong dynamics. We achieve a good approximation for this patient with an average mean squared error (MSE) of 0.054 across the eleven testing scenarios. Due to regularisation and pruning, the number of free parameters (i.e. non-zero weights $w_{f}$) is 40, resulting in a sparse model architecture even for this extreme patient.

Using our transfer learning approach, we successfully learn the other 512 virtual patients corresponding to the defined hypercube of parameter values of *γ*, *MTT* and $E_{max}$ of the semi-mechanistic model. Learning is achieved on the basis of the five training therapy scenarios. The validation therapy scenarios are used to prevent overfitting while the eleven testing scenarios are used to assess prediction performances.

**Figure 5:**
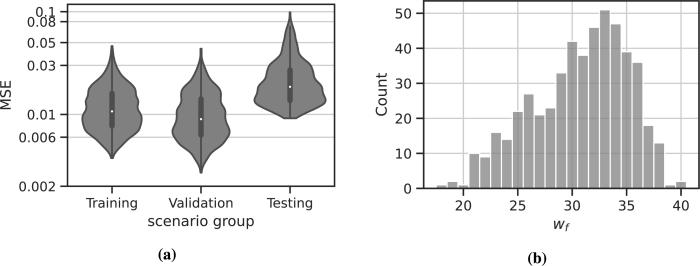
**Results of the transfer learning for 512 virtual patients. (a)** MSE averaged for the different therapy scenarios used for training, validation and testing, respectively. **(b)** Distribution of *w*_*f*_ for the individual networks learned.

We observe a good approximation of the original time series in all instances. The averaged MSEs are 0.012 for the training scenarios, 0.011 for the validation scenarios and 0.023 for the testing scenarios (see also Table 2). As expected, the MSE for the training and validation scenarios are smaller compared to the testing scenarios. The final number of non-zero weights $w_{f}$ ranges in between 18 and 40, with an average of 31. The distribution of the average MSE for scenario groups is displayed in Fig. 5a and the distribution of $w_{f}$ is displayed in Fig. 5b.

**Table 2:** **Population results for network parameters and performance metrics.** We present the average MSE for the different scenario groups and the average *w*_*f*_, as well as the standard deviation *σ* and the value ranges.

*x*	MSE$\;{}_{test}$	MSE$\;{}_{train}$	MSE$\;{}_{val}$	*w*_*f*_
$\bar{x}$	0.023	0.012	0.011	30.7
*σ*_*x*_	0.013	0.006	0.006	4
max⁡(x)	0.32	0.11	0.05	40
min⁡(x)	0.0025	0.0024	0.0028	18

**Figure 6:**
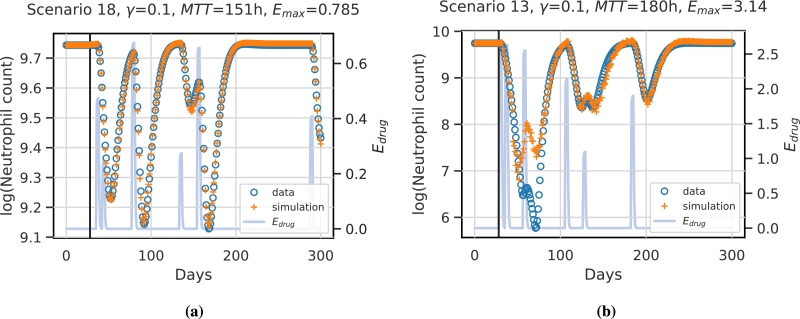
**Example simulations of test scenarios for two different virtual patients/scenarios.** The vertical black line indicates the prediction start. Values before this time point are assumed constant and are used to initiate the network. Data points are derived from the mechanistic simulation and are shown in blue circles. Network prediction is marked by orange crosses. The function representing the therapy effect is displayed in light blue. **(a)** shows the best predicted patient and scenario (scenario 18). The patient is characterised by the parameters *γ* = 0.1,  and *E*_*max*_ = 0.785. **(b)** shows the worst approximation, which corresponds to scenario 13 and patient parameter settings of *γ* = 0.1,  and *E*_*max*_ = 3.14. Note that y-axes differ between the two patients.

Overall, the generalisation performance is good. To illustrate this, we show the prediction performances of two selected patients/scenarios in Fig. 6. While the first patient/scenario is fitted best, shown in Fig. 6a, the second patient/scenario represents the worst fit, shown in Fig. 6b.

The first patient/scenario is characterised by weak chemotherapy effects, i.e. low values of $E_{max}$. Thus, resulting dynamics are mild and are easy to capture by the NARX model. The reason for the bad fit of the second patient/scenario is that the nadir phase after the second and third chemotherapy application is not predicted correctly. This scenario is characterised by a short distance between two strong chemotherapy applications, i.e. the time distance between first and second chemotherapy applications is only seven days resulting in extremely low nadir values which were not observed in the training scenarios. Moreover, the parameter setting of this patient is extreme, i.e. it lies on an edge of the considered parameter hypercube with maximum values for *MTT* and $E_{max}$, while *γ* is minimal. This is also far away from our index patient characterised by a high *γ* value. However, the patients learned model performs well for other therapy scenarios (see supplement, Figure S2). Thus, we conclude that a combination of extreme parameter values and extreme therapy scenarios causes a relevant deviation of prediction and data.

**Figure 7:**
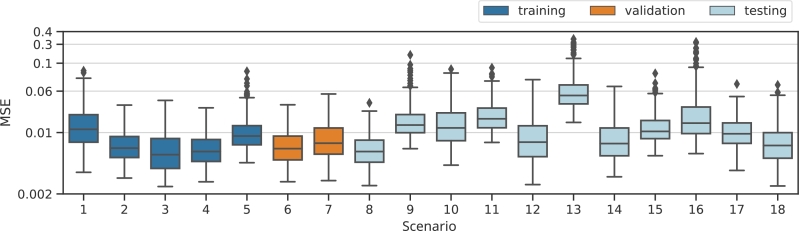
**Box plots of individual MSEs for all scenarios considered.** Scenarios 1-5 are used for training, 6-7 for validation and 8-18 for testing the generalisation performance of the learned individual neural network models.

To investigate this observation systematically, we provide and compare the prediction errors of the learned models between scenarios, see Fig. 7. Of note, the models performed similar for most scenarios. Thus, we conclude that the models generalise well to other therapy scenarios. A possible exception is scenario 13 due to the short distance between the first and the second chemotherapy applications and its high dosages (four times as normal for the first and 3.5 times as normal for the second). Accordingly, we observe the worst fits for this scenario.

### 3.3 Impact of patient characteristics on prediction performance of the individual NARX models

**Figure 8:**
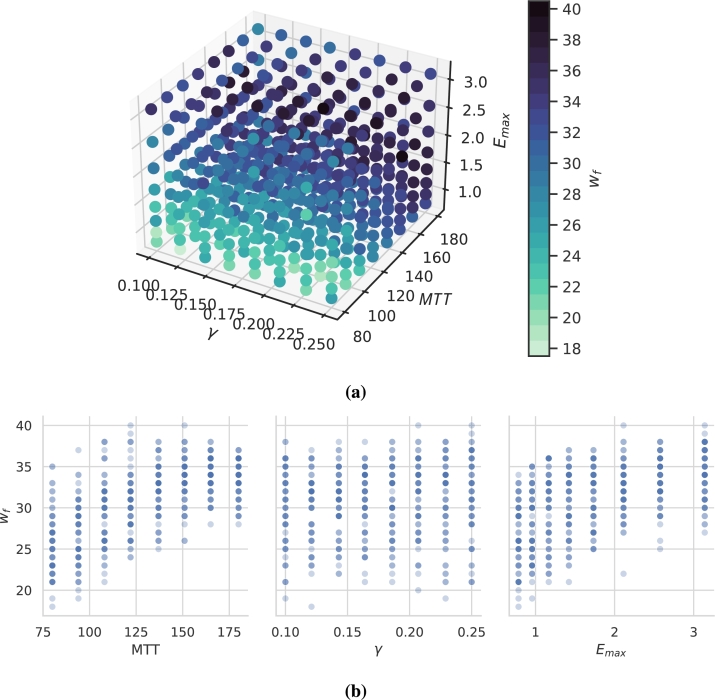
**Number of non-zero weights of learned individual NARX models. (a)** 3D-plot of the relation between patient parameters and *w*_*f*_. **(b)** For better interpretation, we present 2D projections of these relationships. Darkness of points corresponds to the number of parameter settings resulting in the same number of non-zero weights. We observe a correlation between *w*_*f*_ and *MTT* and *E*_*max*_.

Now, we investigate how the individual parameter settings of our virtual patients affect the complexity of the learned NARX model. Complexity is assessed in terms of $w_{f}$. This number ranges in between 18 for the least complex patients and 40 for the most complex patients (see Table 2). The dependence of $w_{f}$ on mechanistic model parameters is displayed in Fig. 8, in 3D in Fig. 8a and for better interpretation as a 2D projection in Fig. 8b. As it turns out, $w_{f}$ is positively correlated with both, *MTT* (Spearman's ρ=0.75) and $E_{max}$ (ρ=0.68) while there is no strong dependence on *γ* (ρ=0.18).

**Figure 9:**
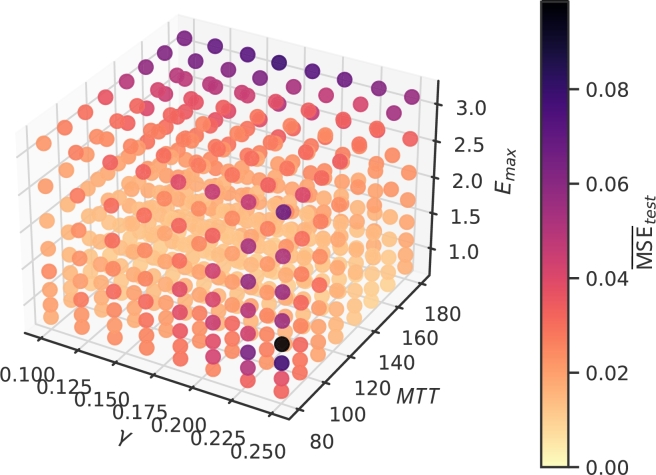
**Average test performance of learned individual NARX models.** 3D-plot of the relationship between patient parameters and average generalisation performance. Two edges with less optimal performance are detectable.

Next we analyse the relationship between prediction performances and model parameters. For this purpose, we average the prediction performance over all testing scenarios per patient. Results are shown in Fig. 9. We do not observe a correlation between model complexity (i.e. number of non-zero weights $w_{f}$) and prediction performance (r=0.002). Prediction performances are largely homogeneous across the considered parameter space. Average performance is only correlated with $E_{max}$ (ρ=0.58), while correlations with *γ* (ρ=0.11) and *MTT* (ρ=−0.08) are small. However, there are two types of parameter constellations resulting in inferior prediction performance, both corresponding to specific edges of the hypercube. One is characterised by a combination of a high toxicity $E_{max}$ and a longer transit time of blood cells *MTT*. These parameter settings imply deep and prolonged nadir phases. The other edge with inferior performance is characterised by a high feedback strength *γ* and a low *MTT*. These parameter settings result in strongly oscillating dynamics, i.e. large differences between days.

### 3.4 Perspective for real world application

We aim at demonstrating how our approach can be applied to real world data. The general idea is to predict an individual's chemotherapy-induced haematotoxicity of a current therapy cycle based on its observational data collected at previous cycles. In analogy to the approach of [9], we here use individual leukocyte data of two cycles of chemotherapy to establish an individual prediction model to simulate the effects of further chemotherapy cycles. Since data of single patients are sparse, we use our transfer learning approach to facilitate the development of an individual NARX network. We demonstrate this approach for two patients (#290 and #760) with high-grade non-Hodgkin's Lymphoma treated with chemotherapy. The data originate from the NHL-B trial [33], [34].

Patients were treated with the chemotherapy regimens CHOP-21 and CHOEP-21, respectively. They received the same dosage of treatment every 21 days over six treatment cycles. CHOP is a treatment consisting of the cytotoxic drugs cyclophosphamide, doxorubicin, and vincristine applied at cycle day one. For CHOEP, the cytotoxic drug etoposide is added at cycle days 1-3.

To apply our NARX framework to these patients, we first fit the Friberg model to the data of the first two treatment cycles of each patient. Then, we simulate our eighteen treatment scenarios using the respective estimated semi-mechanistic parameter combinations. We utilise these simulated data to train and validate the individual NARX networks as for our virtual patient population. Next, we apply our transfer learning approach by retraining the NARX networks based on the real patient data of the first two treatment cycles. Retraining is performed with a low learning rate and static pruning with a pruning cut-off of δ=0.003 to preserve the sparse network architecture and to avoid overfitting of the sparse data of each patient.

**Figure 10:**
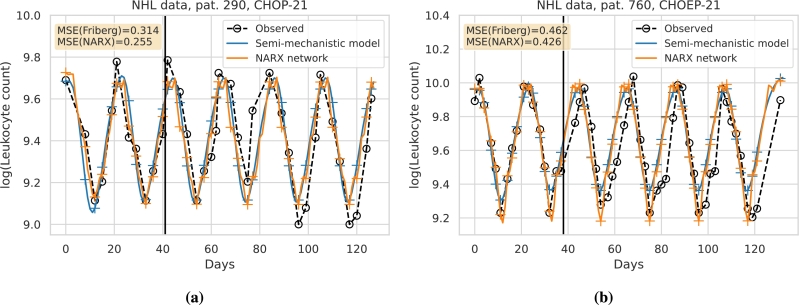
**Comparison of prediction performances of the Friberg model and our NARX framework regarding individual patient therapy courses.** We present the log-scaled time series of leukocyte counts for two patients. **(a)** The patient on the left received six cycles of CHOP-21. **(b)** The patient on the right received six cycles of CHOEP-21. Available leukocyte data are presented as black circles. Models were calibrated on data of the first two therapy cycles (vertical black line). Data on the right of the line were used to test the prediction performance of the models. In blue, we present the results of the Friberg model, with crosses marking time points of possible comparisons of model predictions and patient data. Likewise, the NARX model is shown in orange. For both patients, the NARX network prediction results in lower MSE compared to the Friberg model.

Results are shown in Fig. 10. In Fig. 10a, we present the results for patient #290 and in Fig. 10b, the results for patient #760. The number of free weights after retraining is $w_{f}=29$ for patient #290 and $w_{f}=24$ for patient #760. The prediction performance is presented in terms of the MSE of the patient data for the third to the sixth treatment cycle. It turns out that the MSE is smaller for our NARX model compared to the Friberg model.

## 4 Discussion

Cytotoxic chemotherapy applications often result in strong dynamics of white blood cells. Deep nadir phases are of particular clinical relevance due to an increased risk of life-threatening infections. It is an important clinical problem to predict and ameliorate these side effects. However, due to large patient heterogeneity, this is difficult to achieve at an individual level. Attempts to solve this task comprise statistical risk models [1] or more or less complex (semi-)mechanistic models of haematopoiesis fitted to individual data and used for prediction [3], [4], [5], [6], [7], [8], [9]. Although showing some success, these models are hampered by their predefined and fixed model structure which does not account for the large heterogeneity in complexity of individual time series data.

Here, we aim to establish an alternative machine learning based approach, in particular based on NARX neural networks, to predict individual white blood cell dynamics under chemotherapy. To achieve this goal, we conducted a theoretical study based on virtual patient data. Since real world patient data are typically sparse, another aim was to establish a transfer learning approach, i.e. the network architecture is built on a limited set of data, and then, translated to other patients and treatment scenarios. This closely resembles an envisaged real-world application for which closely meshed or imputed time series data are available for a limited set of patients, only.

We obtained our virtual patient population based on simulations of the semi-mechanistic model of Friberg et al. [3]. This model contains five differential equations and three parameters, the transit time *MTT*, the feedback strength *γ* and the baseline level of white blood cell counts $Circ_{0}$. Chemotherapy actions are modelled by a suppression function of haematopoiesis. We here considered applications of etoposide according to Friberg et al. [3]. Respective nonlinear chemotherapy functions contain two additional model parameters, the maximum therapy strength $E_{max}$ and the chemotherapy concentration $EC_{50}$ resulting in half-maximal strength. We used the same pharmacokinetic model of etoposide concentrations as in Friberg et al. [3]. Other chemotherapies can be modelled analogously.

Despite of the relatively simple structure of the Friberg model, it is able to produce complex and realistic dynamics of white blood cells under chemotherapy [3]. Patient heterogeneity was mirrored by assuming a range of possible values for two of the three parameters of the basic model and the maximum therapy strength $E_{max}$ as well. The parameter $Circ_{0}$ was assumed constant since data were scaled prior to NARX modelling. Likewise, the second parameter of the chemotherapy function $EC_{50}$ was set constant due to low sensitivity.

We generated treatment scenarios by varying the distances and doses between a total of six etoposide applications. In order to test the performance of our approach, we deliberately allowed extreme scenarios, e.g. with short distances between two intense chemotherapy courses, such as scenario 13. In clinical practice, such intense scenarios are typically accompanied by additional supportive care such as G-CSF treatment, which we did not consider here. All virtual patients were simulated with the same generated treatment scenarios for comparability. Cell counts were calculated on a daily scale to increase realism of the virtual data. We simulated a total of five training, two validation and eleven testing scenarios to increase learning speed and to assess prediction performance for a broad range of therapy scenarios.

Correctly predicting nadirs of cell counts is particularly important for clinical application. Thus, we logarithmized all cell counts. Another advantage of this logarithmic scaling is the increased sensitivity of the sigmoid activation function, which we choose in our NARX framework. The sigmoid function is commonly used in network models for time series forecasting, but introduces the possibility of lacking sensitivity with respect to extreme values. For our virtual data, we observe only one parameter and therapy setting, where the nadir was not correctly predicted (scenario 13, parameter setting γ=0.1, , $E_{max}=3.14$). We conclude that the sigmoid activation function works well for our problem.

For transfer learning, we selected an index patient with extreme parameter values, assuming that learning respective data requires the highest number of non-zero weights of the network. The resulting network structure served as a basis to learn the other patients. In principle, individual learning attempts are possible but much more time-consuming. This approach worked well in our hands, since virtual patients far away from the index patient could be learned successfully. Thus, we suppose that the approach could be translated to real-world settings vastly increasing learning speed and ameliorating over-fitting.

Considering the testing scenarios, we observe good generalisation for the virtual patient population, even for extreme semi-mechanistic parameter settings and therapy scenarios. The resulting mean squared errors are small compared to the natural variation of blood cell counts [35], [36], [37], i.e. the prediction performance is sufficient to qualify for real-world applications.

Prediction performances differ between patients. We identify two clusters of parameters with inferior prediction performance. One cluster is characterised by large *γ* and small *MTT* resulting in stronger dynamics, which are insufficiently captured by the chosen time distance of one day between measurements. This might be improved by more closely meshed sampling schemes, which however, are unrealistic in clinical settings. The other cluster is characterised by large $E_{max}$ and large *MTT*, resulting in prolonged nadir phases. Here, our transfer learning approach might be too restrictive. Indeed, we observe a positive correlation between the number of non-zero weights $w_{f}$ and the parameters $E_{max}$ and *MTT*. Moreover, these patients receive higher weights to later memories. Thus, fine-tuning of these patients by reactivation of pruned weights could be considered.

Patient heterogeneity is also reflected by the number of non-zero weights. Of note, model complexity and test performance are uncorrelated indicating that weights are selected in a parsimonious manner and in dependence on patient complexity avoiding over-fitting. Model performance depends more strongly on the therapy scenario, i.e. the complexity of the administered treatment plan.

To provide a perspective for a real world application of our NARX modelling and transfer approach, we provide an example of predicting individual haematotoxicities of two patients. Both patients received a therapy not directly considered in our simulation framework but with a similar scheduling structure and without extensive additional medications. Fitting the semi-mechanistic Friberg model already resulted in a good agreement of simulation and data. Thus, our simulation study can be considered valid for this situation. We applied our NARX modelling based transfer learning approach and demonstrated that it resulted in improved prediction performances compared to the original Friberg model. Further improvements can be expected if considering drug specific pharmacokinetic effects. A more comprehensive comparison with other modelling frameworks and approaches and including a larger patient population is planned for the near future.

We conclude that we successfully implemented a NARX-based approach to learn a family of dynamical systems intended to describe individual patient time series data of white blood cells under chemotherapy. Our model reduction methods in combination with our transfer learning approach proved to be successful to avoid over-fitting with prediction errors smaller than the natural variation of white blood cells. In the future, we will examine the suitability of our approach on complex real-world data and compare performances with existing mechanistic models.

## CRediT authorship contribution statement

**Marie Steinacker:** Performed the experiments, Analyzed and interpreted the data, Wrote the paper. **Yuri Kheifetz:** Conceived and designed the experiments, Analyzed and interpreted the data. **Markus Scholz:** Conceived and designed the experiments, Analyzed and interpreted the data, Wrote the paper.

## Declaration of Competing Interest

The authors declare the following financial interests/personal relationships which may be considered as potential competing interests: M. Scholz received funding from Pfizer Inc. for a project not related to this research.

## Appendix A Supplementary material

MMC The captions for the supplementary sections are as follows: S1 Flowchart of analysis: We present the analysis pipeline applied to our virtual patient data. S2 Treatment scenarios: We present the code for generating our virtual patient data. S3 Performance plots extreme virtual patient: We present the performance plots for all treatment scenarios for the virtual patient with an extreme mechanistic parameter setting. MMC
