# Supplementary material for: Individual modelling of haematotoxicity with NARX neural networks: A knowledge transfer approach
Source: Heliyon. 2023 Jul 5;9(7):e17890. doi: 10.1016/j.heliyon.2023.e17890 (PMC10362198; doi:10.1016/j.heliyon.2023.e17890)
Supplement: MMC — The captions for the supplementary sections are as follows: S1 Flowchart of analysis: We present the analysis pipeline applied to our virtual patient data. S2 Treatment scenarios: We present the code for generating our virtual patient data. S3 Performance plots extreme virtual patient: We present the performance plots for all treatment scenarios for the virtual patient with an extreme mechanistic parameter setting. [file mmc1.pdf]

Supplemental material

S1. Flowchart of analysis

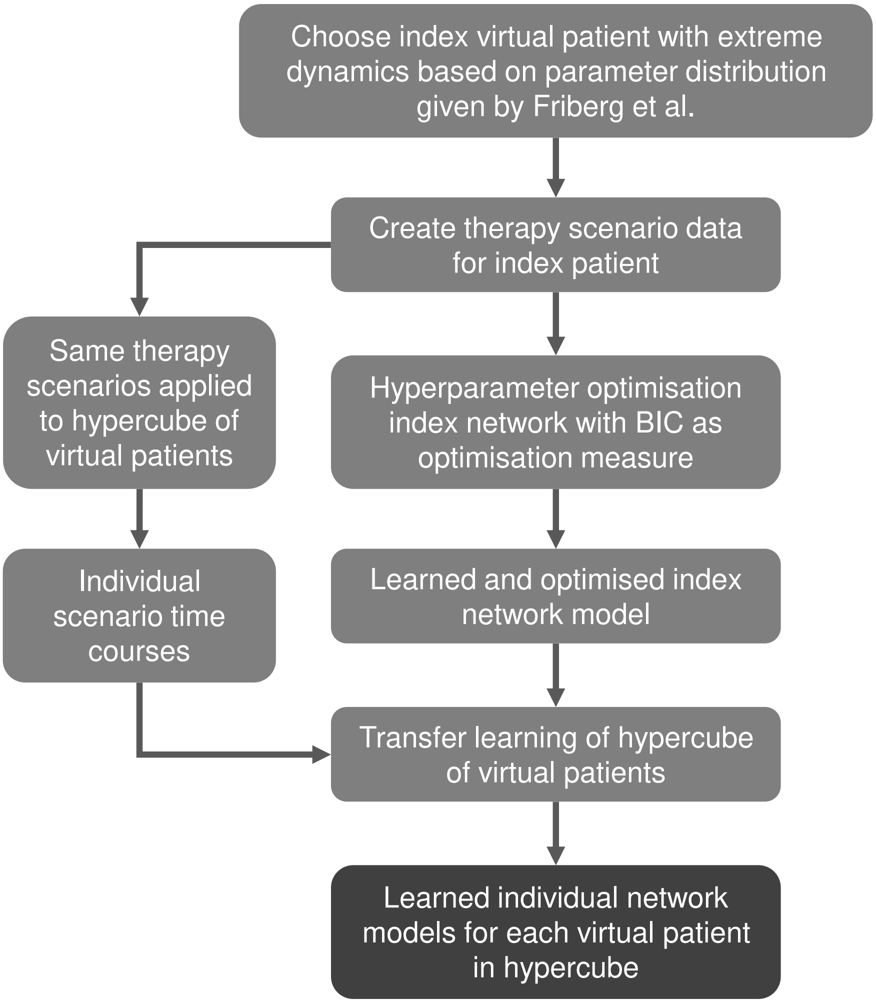

Figure S.1: Flowchart of approach to individualised network models for patient population

S2. Treatment scenarios

S2.1. Code for scenario creation

```
seeds (therapy_dates , dosages):
array([[3356737, 4913634, 2965200, 2875040, 1800356, 1683633,
1135631 684210, 1308554, 6687090,  298367, 1824443, 6615170,
4987257, 6772834, 6741566,  861091, 1145566],
[4696088, 9284172, 924207, 4938981, 9804438, 4656330, 7243077,
44631, 7075963, 8497091, 4577874,  838371, 7822484, 4966962,
818217, 7420609, 4643876, 1609814]])

code:
# storage array
therapies =  np.zeros((10,2,6))
# possible ratio values
geom = np.around(np.geomspace(0.25, 4.,17, endpoint=True), 2)
# fill storage array
for i in range(10):
    # therapy days
    np.random.seed(seed[0,i])
    therapies[i,0] = np.sort(np.random.choice(np.arange(4,43) * 7,
    6, replace=False))
    # dosage
    np.random.seed(seed[1,i])
    therapies[i,1] = np.random.choice(geom,6, replace=False)
```

Table S.1
Treatment scenarios: treatment administration day refers to the number of days after the start of observation, where treatment is administered. The dosage refers to the ratio of the standard dosage of 375 mg/m^2. Treatment days and dosages are drawn randomly with constraints, see code implementation.

	Scenario	Therapy Administration Day<br>Dosage					
Training	1	49	63	91	140	266	280
		0.59	2.83	1.19	0.35	3.36	0.50
	2	84	105	133	189	210	245
		0.71	1.41	0.35	0.84	4.00	3.36
	3	35	56	84	105	154	161
		2.38	3.36	1.19	2.00	0.71	0.84
	4	42	77	168	189	231	252
		0.71	0.25	2.38	1.19	0.42	1.00
	5	70	161	168	203	210	273
		0.71	0.42	1.68	2.00	1.19	0.84
Validation	6	56	84	98	147	210	280
		0.59	0.50	2.83	0.25	1.41	1.19
	7	28	42	91	189	224	231
		1.41	1.19	0.35	2.83	0.25	3.36
Testing	8	35	77	112	119	210	266
		0.71	4.00	0.30	2.00	1.68	0.42
	9	35	56	63	154	259	287
		0.50	4.00	0.84	0.35	0.59	2.83
	10	35	84	98	161	252	294
		0.50	3.36	1.00	1.41	0.25	2.38
	11	49	77	84	119	210	231
		0.42	1.68	2.83	2.00	0.35	0.84
	12	182	189	231	245	266	280
		0.84	0.71	0.50	2.00	0.42	4.00
	13	28	35	56	105	126	182
		4.00	3.36	2.83	1.41	0.35	1.00
	14	35	119	140	154	175	182
		0.84	4.00	0.25	0.71	0.35	0.59
	15	49	105	119	126	154	273
		2.83	0.30	4.00	0.42	1.41	0.59
	16	56	126	231	252	259	273
		1.00	0.59	0.50	4.00	0.71	0.30
	17	28	77	126	133	168	252
		0.50	2.83	0.59	3.36	4.00	0.42
	18	35	42	77	133	154	287
		1.00	0.30	4.00	0.35	3.36	0.71

S3. Performance plots extreme virtual patient

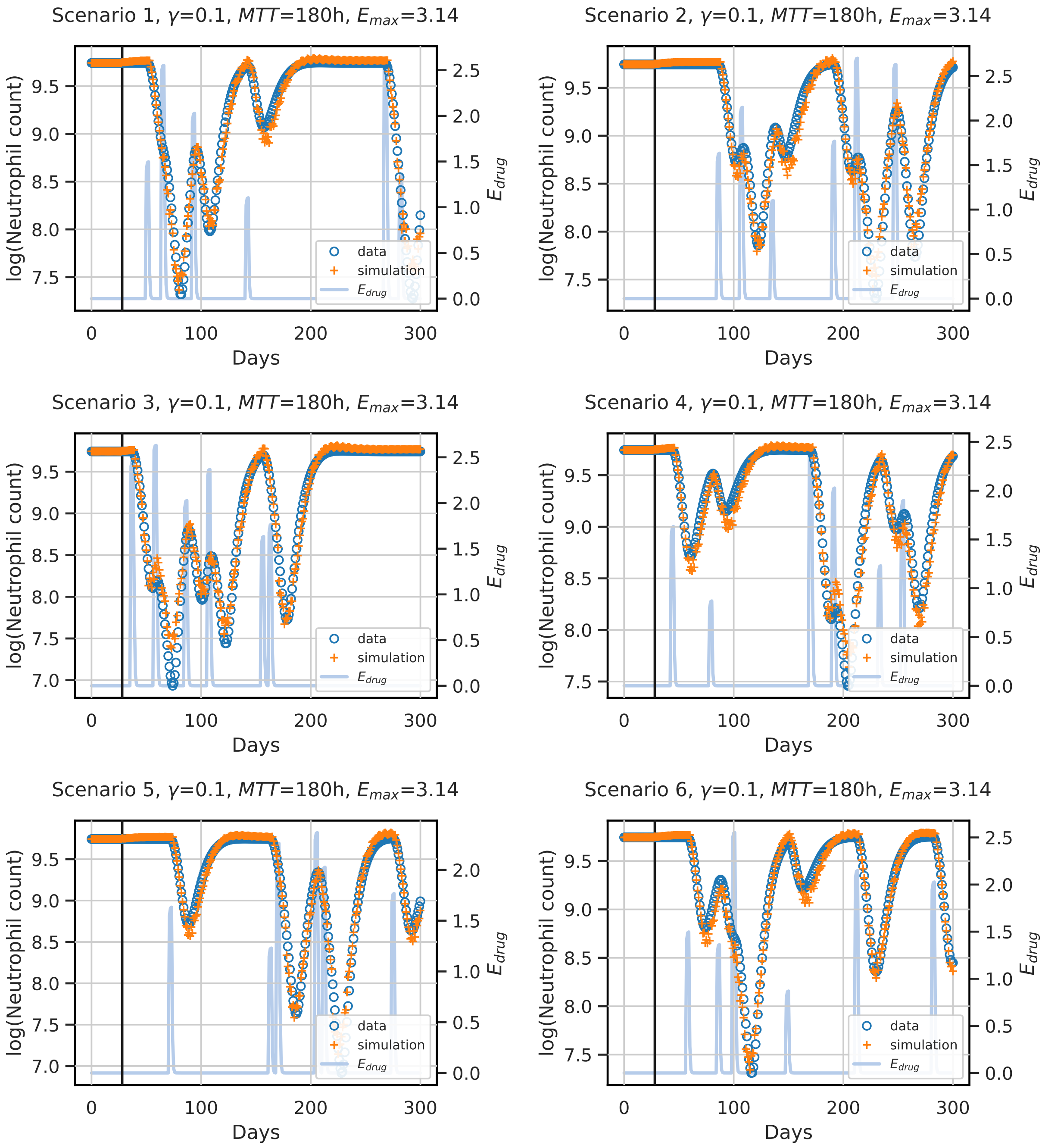

Figure S.2: Scenarios 1-6

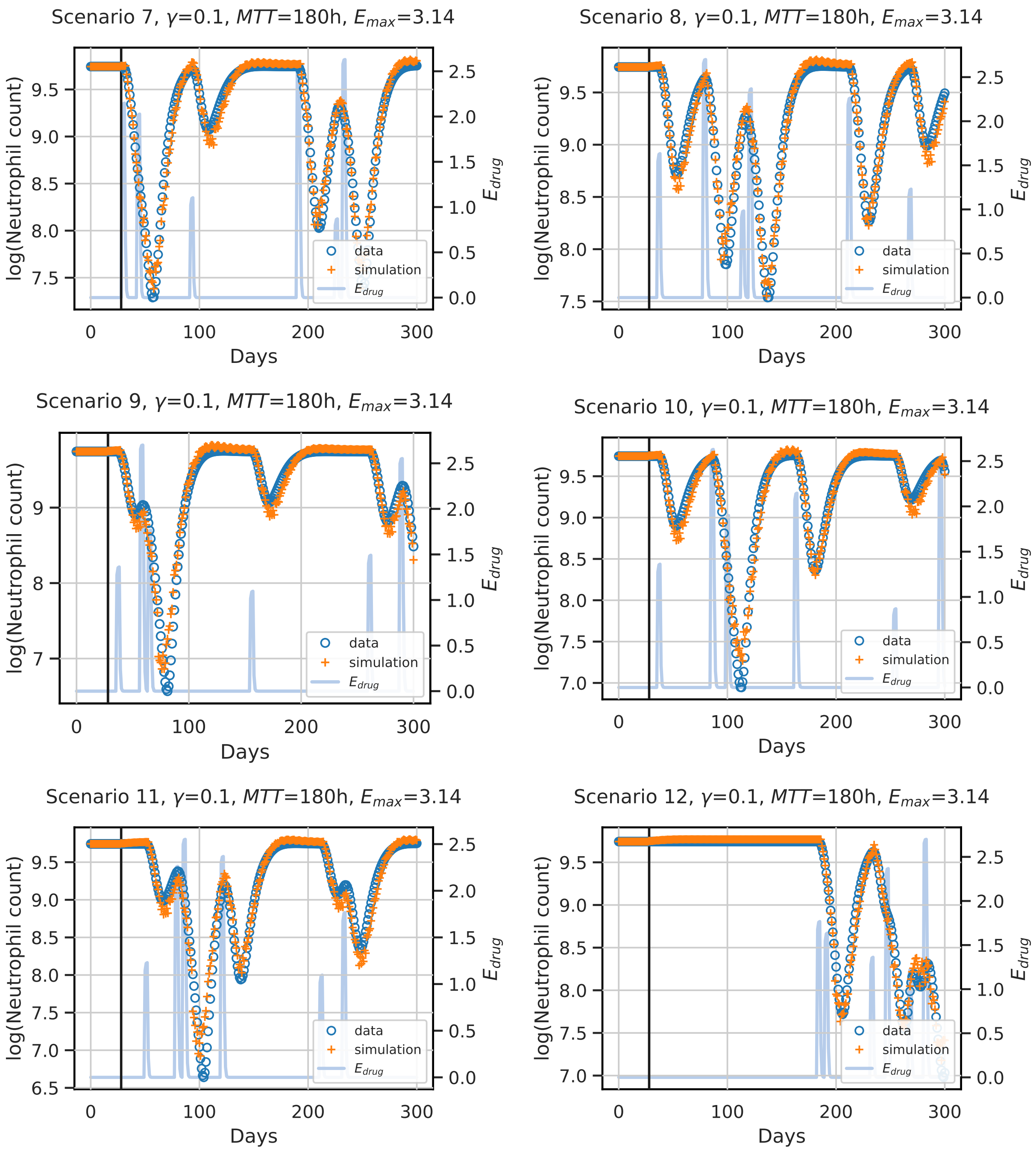

Figure S.2: Scenarios 7-12

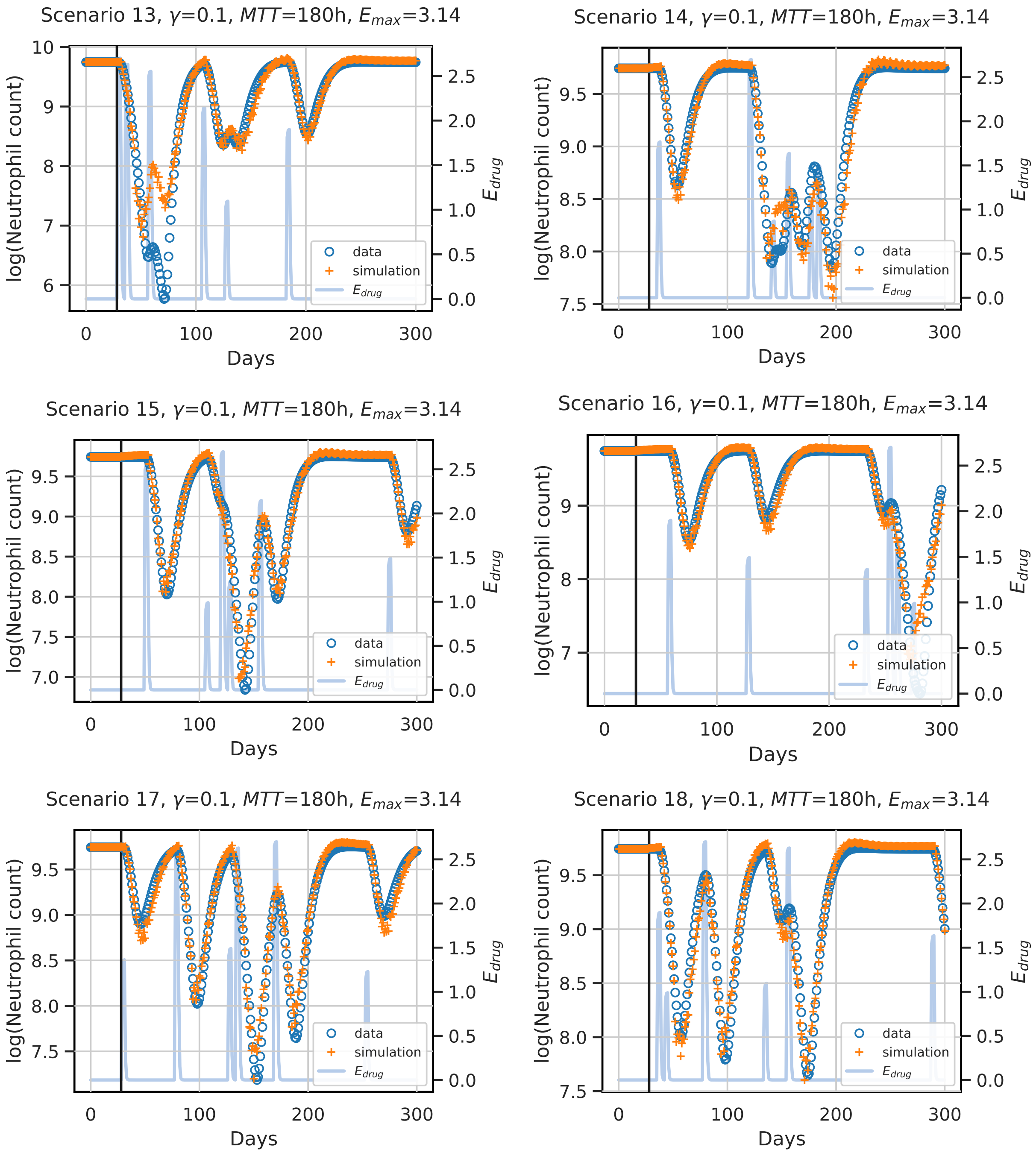

Figure S.2: Scenarios 13-18
